# Passing Events and Topics

**Published:** 1921-12

**Authors:** 


### PASSING EVENTS AND TOPICS.

GILBERTIAN possibilities are suggested by the announcement that Bavaria is to legislate against gluttons. We are not told what is to constitute gluttony within the law, nor whether the offence is to lie in excess of quality or of quantity. We picture the Restaurant Inspector, with his callipers and portable weighing-machine, operating on each customer before and after dinner, and his yet more terrible assistant in plain clothes, alert for too visible (or audible) appreciation of the fare. And what of the criminal who incites to gluttony? Fine and imprisonment may attend the friendly waiter who suggests that so-and-so is very nice to-day; the affectionate wife who would persuade her spouse to eat a little bit more will do so at her peril.

---

CLOSE upon eighty per cent. of the cases which enter the maternity department attached to King Edward VII. Hospital, Cardiff, come from the Welsh metropolis. At present the city authorities pay all the expenses involved in cases sent to the institution on the authority of the medical officer of health, but these account only for ten per cent. of the number. In view of the hospital's financial position, it is appealing for more adequate support from all quarters where its benefits are received, and in pursuance of this policy Sir William Diamond, Chairman of the Board of Management, and Dr. Ewen Maclean appeared before the Cardiff Maternity and Child Welfare Committee. There are thirty-one beds for mothers and twenty-five cots for children in the maternity wards of the hospital, and the cost per patient per day has now fallen to 11s. The admissions so far this year have numbered 417, but unless more support is forthcoming Sir William Diamond intimated that they would find it very difficult to carry on the work. Cardiff is not alone in getting from the maternity department of its hospital far more benefit than it pays for, and hospitals in other towns which are anxious to put their houses in order might with advantage make similar appeals to their civic authorities.

---

THE Urban Council of the colliery district of Maesteg has achieved the distinction of being the first in South Wales to equip and open a maternity hospital. Like most other urban areas in industrial Wales, Maesteg found that the serious overcrowding and unsuitability of the dwellings occupied by the working people demanded the provision of such an institution. A substantial private residence was acquired by the Child Welfare and Maternity Committee of the Council, which has equipped it with eight beds and up-to-date provision for the work intended. The hospital was formally opened by Mr. T. E. Hopkins, J.P., Chairman of the Child Welfare and Maternity Committee, when Dr. Walter Kirby, the Medical Officer, congratulated Maesteg on its pioneering enterprise.

THE statistical statement of the present position of the London hospitals was given recently by Sir Alan Anderson in an address to the Charity Organisation Society. As honorary secretary to King Edward's Hospital Fund for London, Sir Alan had been set during the summer to estimate the total deficiency for the current year. The estimate was £500,000, or the equivalent of the total grant given by the Government for the hospitals of the whole country. The least solvent London hospitals had an estimated deficiency of some £230,000 this year, which implied a running into debt of £20,000 a month, a fact which the Fund had reported to the Hospitals Commission. Unless some great change for the better occurred a large number of beds would have to be closed. Since 1913 hospital expenditure in London had risen from £1,188,000 to £2,841,000, and the income from £1,478,000 to £2,447,000, of which last charity in 1920 had supplied £1,868,000 and patients or prospective patients £272,000.

---

IN towns with a reasonably good public health administration the medical officer of health knows every morning at his office in what part of his district are cases of infectious diseases. The morning post brings the notification, or cases are notified informally by doctors over the telephone. Certain of these notified cases, say of scarlet fever, are in good-class houses, where the inhabitants are not overcrowded; and in such instances the patients can very well remain at home. In other instances, however, the home accommodation is insufficient, and the case can properly be removed to hospital. It is the medical officer of health who decides which case is to go to hospital and which is to stay at home. In London, apparently, or at any rate in some parts of the Metropolis, the question of the admission of patients to isolation hospitals is not decided by the medical officer of health. He has no say in the matter. The doctor or the head of the infected family rings up the Metropolitan Asylums Board and the case is removed whether it is or is not a suitable one to go to hospital. We are glad to hear that this slovenly practice is to be discontinued, and that in future the cases of infective disease will be sent into hospital only upon the recommendation of the metropolitan medical officers of health.

---

It is justifiable to make use of experimental findings in the interpretation of clinical phenomena, and on this basis it may be concluded that the function of antitoxin formation in diphtheria varies in different individuals both with regard to speed and quantity. We may assume that the morbid processes reach their climax and commence to decline as soon as antitoxin appears in the circulation, for from this moment newly formed toxin will be neutralised. It is, therefore, clear that the symptoms of an infection of a given virulence will be milder the greater the speed and abundance of antitoxin formation. Thus, the most severe manifesta-

**PASSING EVENTS AND TOPICS—(*cont.*).**

tions will be encountered in those individuals who are unable to produce it altogether or only after a long delay. In practice children are found to exhibit all grades of reaction between the two extremes. The mildest cases may be expected who did in fact have traces of antitoxin before the onset of the illness, though the amount in circulation was too small to be detected clinically and insufficient to prevent infection. Such children would be especially adapted to react to the stimulus of circulating antitoxins by producing the necessary quantity of antibodies in the shortest space of time. Thus the conclusion is reached that, in addition to the local protection derived from the intact and impregnable mucous membrane, there are two factors producing immunity against diphtheria : the one is humoral in nature and its presence is determined by demonstrating and measuring specific antibodies in the serum; the other is the cellular factor, which is based on the varying faculty of the cells to generate antitoxins. Humoral immunity signifies absolute protection as long as antibodies are circulating. Cellular immunity, while unable to prevent infection, determines the intensity and duration of the attack. The later and the less efficiently the defensive mechanism of cells reacts to the morbid stimulus, the later the effects of the organisms and their toxins can be counteracted, and, naturally, the more opportunity is given for the diphtheria bacillus to develop its dangerous activity. In this way we are able to determine the malignant forms of diphtheria, which are due to the complete inability of the body to defend itself against the toxins of diphtheria.—" New York Medical Record."

---

In the words of a well-known resident, the Leicester Infirmary ." week " should have been a " fortnight," for toward the close everybody was talking Infirmary. This does not imply a lack of interest in the early events—on the contrary. At the service at St. Martin's, the same building where, exactly 150 years ago, the inaugural service of the institution was held, the Archdeacon of Leicester gave a send-off to the week's festivities. The Archdeacon offered his special tribute to the nursing staff—represented by the Matron (Miss Vincent) and about fifty nurses. Two receptions by the Chairman of the Board and Mrs. Fielding Johnson were social successes. Some 700 friends enjoyed hospitality on each occasion, and the entire number left with a deepened interest in the work of the institution.

A pageant, presented by Mr. Herbert Pochin and his friends, was perhaps the most successful effort of the week. The 200 players enjoyed their individual parts as much as the audience (upwards of 2,000) enjoyed the entertainment. Mr. Herbert Pochin's zeal was caught by every member of the cast, who depicted Simon de Montfort's visit to Leicester on May 3, 1239, to grant quit claim of his rights over the Cowhay Pasture to the Burgesses of the town. The week closed with a public meeting, when the speakers carried their audience back to 1771 in their endeavours to contrast the position of the town at that time—without a hospital or even a workhouse infirmary—with to-day. Sir Arthur Hazlerigg, Bart., a cheerful speaker, occupied the chair. A paper by Mr. C. J. Billson, M.A., on " Dr. Watts, the Original Founder of the Infirmary," gave evidence of a great deal of research which will be of future interest, and Colonel C. J. Bond, in his paper on " The Place of the Hospital in the Life of the Community," urged that " Hospitals largely in the past have been centres for the treatment and cure of disease. In the future they will become more and more centres for the diffusion of knowledge concerning the cause and the way to prevent disease." But to carry out this greater service he urged more doctors, more beds, more nurses were necessary.

---

In a report from San Francisco, dated June 16, 1921, an account is given of the occurrence of nine cases of typhoid fever among the members of the crew of the steamship " Lake Gunni," which evidently originated in a carrier who was a member of the crew.

The carrier (H. L.), a fireman, was admitted to the hospital for observation on May 30, and the typhoid bacillus was demonstrated in his urine. He informed the medical officer in charge that he had had typhoid fever in New Orleans, where he was discharged from the hospital on April 26, and had shipped on the " Lake Gunni " on the following day. The nine cases from the vessel were all admitted to the hospital between May 25 and June 6.

The vessel was visited by a medical officer of the Public Health Service, who examined the water and ice with negative results. The information secured from each of the patients suggested no other common source of infection; and so there seems to be little doubt that this group of cases originated in the typhoid bacillus carrier.

---

Those who themselves have enough to eat are inclined to discuss in trains and newspapers whether they can or will or should help those who are reported to be starving in other countries. They would do something at once if they saw a starving child in the road, but Russia is so far away, and, moreover, Russia has been possessed by a dangerous political creed which might spread to other countries. The waverer who is untouched by the prospect of happiness, now and in the future, to be derived from feeding the hungry and clothing the naked may bear in mind that cholera and typhus which decimate famine areas are no respecters of persons, and will if unchecked spread West; while Russia, when happy and prosperous again, will be a great market for British goods.—" Maternity and Child Welfare."

---

Modern epidemiologists have many questions to answer before their studies and hypotheses can truly be considered as an exact science. Leprosy was once endemic in this country, and it is here no longer. Plague was endemic and epidemic in these islands from 1348 to 1667; it came suddenly, and as suddenly departed. Smallpox and scarlet fever are changing their types. Influenza seems to be in a perpetual flux; during a few years it causes terrible mortality, and at other times it is a trivial illness; its association with the neurotoxic group of diseases such as encephalitis is still undetermined. These and many other riddles are awaiting the solution of the thinker. Among the few men who can rightly claim to be called epidemiologists we should rank the medical officer of health to the London County Council. Dr. Hamer has the great virtue that he is heterodox; and, indeed, that is a virtue to-day when to be respectable is to be conventional, even in thought and in theory. Dr. Hamer, like Creighton, would have little use for the Cathay business. He would not be content to trace a case of enteric fever back to an oyster and to worry no more about it. Nor is he content to accept on the present evidence much of the epidemiology of our times. —" The Medical Officer."

